# The Role of IL-22 in Viral Infections: Paradigms and Paradoxes

**DOI:** 10.3389/fimmu.2016.00211

**Published:** 2016-05-30

**Authors:** Silvia Gimeno Brias, Gabrielle Stack, Maria A. Stacey, Alec J. Redwood, Ian R. Humphreys

**Affiliations:** ^1^Institute of Infection and Immunity, Cardiff University, Cardiff, UK; ^2^Systems Immunity University Research Institute, Cardiff University, Cardiff, UK; ^3^The Institute for Immunology and Infectious Diseases, Murdoch University, Murdoch, WA, Australia

**Keywords:** viruses, cytokines, pathology, immune system diseases, cytomegalovirus

## Abstract

Interleukin-22 (IL-22) is a member of the IL-10 family of cytokines. Hematopoietic cells express IL-22, and this cytokine signals through the heterodimeric IL-22 receptor expressed by non-hematopoietic cells. A growing body of evidence points toward a role for IL-22 in a diverse array of biological functions ranging from cellular proliferation, tissue protection and regeneration, and inflammation. In recent years, the role that IL-22 plays in antiviral immune responses has been examined in a number of infection models. Herein, we assess our current understanding of how IL-22 determines the outcome of viral infections and define common mechanisms that are evident from, sometimes paradoxical, findings derived from these studies. Finally, we discuss the potential therapeutic utility of IL-22 manipulation in the treatment and prevention of viral infections and associated pathologies.

## The IL-22–IL-22R Pathway

Interleukin-22 (IL-22) is a member of the IL-10 family of cytokines that was originally identified as an IL-9-inducible gene produced by mouse T cells (1, 2). Since its discovery, IL-22 expression by a number of immune cell subsets has been detected, including activated natural killer (NK) cells, NKT cells, neutrophils, γδ T cells, innate lymphoid cells (ILCs), and CD8^+^ T cells (3–9). In addition, IL-22 is expressed by CD4^+^ T cells of the T_H_17 (4) and T_H_1 lineage (10). Moreover, T_H_22, a CD4^+^ T cell subset distinct from T_H_1 and T_H_17 cells, has been described (11). T_H_22 cells produce IL-22 independently of IFN-γ and IL-17 production and can be distinguished from T_H_17 cells by the expression of CCR10 (11–14). Given the diversity of the innate and adaptive cells that can produce IL-22 and plasticity among T helper cell subsets, there have been significant efforts to identify common regulators of leukocyte IL-22 production. Subsequently, IL-23 and aryl hydrocarbon receptor (AhR) have emerged as key inducers of IL-22 production in NK cells, ILCs, CD4^+^ T cells, and γδ T cells (6, 15–23).

The IL-22 receptor (IL-22R) is a heterodimer that is composed of IL-22Rα and IL-10Rβ (24). IL-22Rα also forms a complex with IL-20Rβ, which is an alternative receptor complex for IL-20 and IL-24, and is thought to induce signals and biological effects similar to those elicited by the IL-22 receptor complex (25, 26). IL-10Rβ is ubiquitously expressed by most cell types, whereas the expression of IL-22Rα, which ultimately determines the target sites of IL-22, is restricted to non-hematopoietic cells, predominantly epithelial cells of the skin, lung, small intestine, kidney, colon, liver, and pancreas (24, 27). IL-22 binding to the dimeric IL-22R triggers phosphorylation and subsequent activation of the kinases Jak1 and Tyk2, which leads to the activation of the transcription factor STAT3, and also STAT5 and STAT1. Furthermore, MAP kinase and p38 pathways are involved in downstream IL-22R signaling (27, 28). In addition, a soluble, secreted single-chained IL-22-binding receptor, IL-22 binding protein (IL-22BP), can bind IL-22 with stronger affinity than membrane-bound IL-22R, thus antagonizing IL-22 signaling (29–31). IL-22BP expression has been reported in multiple tissues, including the spleen, lung, skin, and female reproductive system (29, 31), and by several hematopoietic cells including immature dendritic cells (DCs) and eosinophils (32–34). IL-22BP is highly expressed in steady-state conditions and downregulated in response to inflammasome activation during tissue damage, coinciding with IL-22 expression, thereby reducing the suppression that IL-22BP exerts on IL-22 signaling (34).

The IL-22–IL-22R pathway exerts a broad array of biological effects in different systems. Experimental models have shown that IL-22 prevents tissue destruction and mediates regeneration of damaged tissue by inducing expression of genes regulating proliferation, survival, and wound healing, ameliorating tissue damage in colitis (35, 36), hepatitis (37, 38), and lung fibrosis (39). Paradoxically, in certain contexts, IL-22 can promote inflammation. For example, overexpression of IL-22 has been associated with psoriasis (5, 40, 41), inflammatory bowel disease (42), and arthritis (43, 44).

The role of IL-22 in tumor development has been reported in several types of cancers, including gastric, lung, colon, hepatocellular, and pancreatic carcinoma, where studies have shown upregulation of IL-22 by tumor-infiltrating lymphocytes in the tumor microenvironment, in addition to the expression of its receptor on cancerous cells (45–49). In hepatocellular carcinoma, pancreatic cancer, and colorectal cancer, IL-22 expression positively correlated with tumor growth, metastasis, and tumor stages (46, 47, 50, 51). This was associated with STAT3 phosphorylation and upregulation of downstream genes Cyclin D (proliferation), Bcl-xl (cell survival), and VEGF (metastasis) (46, 47). Furthermore, recent studies using mouse models of colon cancer have shown that IL-22 produced by CD4^+^ T cells acts upon cancer cells to activate STAT3 and promote the expression of the histone 3 lysine 79 (H3K79) methyltransferase DOT1L, which induces key cancer stem cell genes that contribute to tumor progression (34, 52, 53). Additionally, in colorectal cancer and lung cancer cells, IL-22 contributes to resistance to chemotherapy by activating STAT3 and subsequently upregulating antiapoptotic genes (48, 54).

Interleukin-22 plays an important protective role in host defense responses during bacterial infections. For example, IL-22R signaling increases the production of antibacterial peptides and proteins (27, 40), acute-phase proteins (2, 42), mucins (35, 55), and increases the production of neutrophilic granulocyte-attracting chemokines [as reviewed in Ref. (56)].

The impact of IL-22R signaling extends beyond pathogenic bacterial infections. IL-22 also influences host resistance to bacterial pathogens through regulation of the interface between epithelial cells and microbiota. Germ-free mice exhibit reduced numbers of intestinal ILCs that express IL-22 (57). Tryptophan metabolites produced by intestinal microbiota activate the AhR in ILC3s to produce IL-22, contributing to the containment of commensal bacteria, limiting inflammation, and preserving gut immune homeostasis (58, 59). In return, IL-22 regulates the gene *Fut2* that encodes the enzyme α1,2-fucosyltransferase that catalyzes the addition of fucose residues to glycoproteins on epithelial cells. This influences the nutrient environment of the microbiota and thus impacts on the diversity and composition of the gut flora and subsequently prevents colonization of pathogens (60–62). Indeed, defective fucosylation has been associated with increased susceptibility to candidiasis (63) and the opportunistic pathogen *Enterococcus faecalis* (61). However, IL-22 favors *Salmonella* infection by inducing antimicrobial proteins that sequester metal ions allowing *Salmonella*, which can overcome metal starvation, to outcompete other commensals (64). Thus, context is critical in determining antimicrobial or pathogenic function of IL-22.

Interleukin-22 also contributes to protective immunity in the early stages of fungal infection with *Candida albicans* (65, 66), *Aspergillus fumigatus* (67), and *Rhizomucor pusilluscan* (68). In candidiasis, IL-22 is produced by innate (DCs and CD3^−^ NKp46^+^ cells) and adaptive (T_H_17 and memory *C. albicans*-specific IL-22^+^CD4^+^ cells) immune cells (65, 66), with IL-23 regulating IL-22 production by T_H_17 cells (66). IL-22 targets epithelial cells to release S100A8 and S10A9 peptides that participate in antifungal protection (66). In *A. fumigatus* infections, β-glucan recognition *via* Dectin-1 as well as IL-23 induces lung IL-22 production for antifungal protection (67). Thus, IL-22 orchestrates immune responses to bacterial and fungal pathogens directly, and through the regulation of the intestinal microbiota.

## IL-22 Production During Viral Infections

Although the role that IL-22 plays in bacterial and fungal infections is reasonably well-defined, a picture of how IL-22 functions in viral infections is still being constructed. Experiments using IL-22 fate-tracker mice have demonstrated IL-22^+^ cells in this model are predominantly ILCs, γδ T cells, and CD4^+^ T cells in the gut, skin, and lung under homeostatic conditions (69). IL-22 reporter mice also highlighted the lamina propria as a rich source of IL-22^+^ T cells in steady state (70). However, it is clear that upon viral exposure, IL-22 is produced by a number of leukocytes in response to a broad array of virus infections. For example, pulmonary NK cells produce IL-22 in response to influenza infection (71). IL-23 stimulates the production of IL-22 during bacterial infections (22, 72), and IL-22 expression by pulmonary NK cells is induced by IL-23 *in vitro* (71). Furthermore, influenza induces IL-22 expression by invariant NK T cells in manner dependent upon triggering of the viral RNA sensors TLR7 and RIG-I in DCs and subsequent production of IL-1β and IL-23 (73).

**Figure 1:**
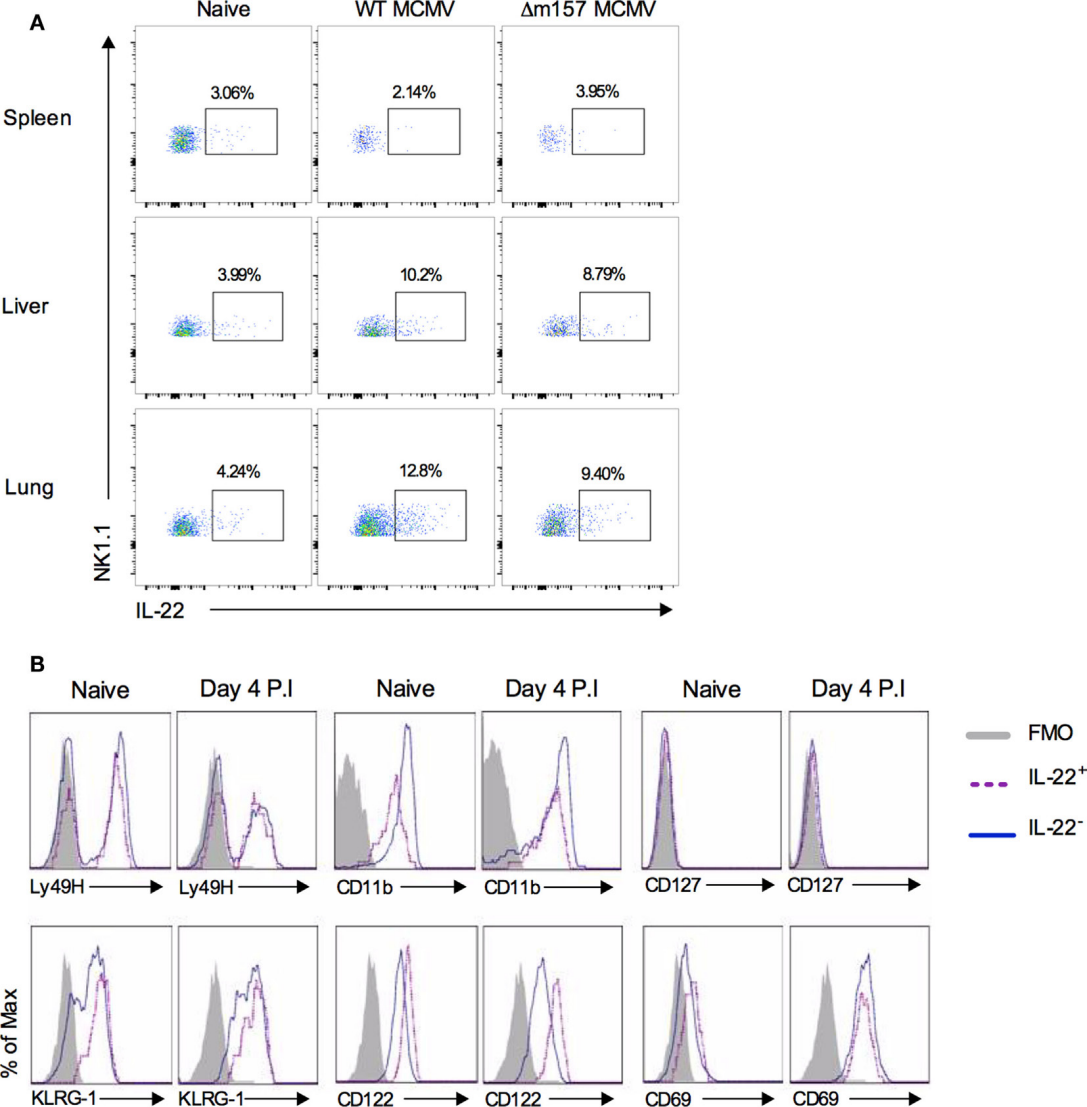
**IL-22 production by NK cells during murine cytomegalovirus infection**. **(A)** C57BL/6 mice were infected or not with 5 × 10^5^ pfu of wild-type (pARK25) or m157 knock out (Δm157) K181 strain MCMV. At day 4 postinfection, spleen, liver, and lung were harvested, leukocytes isolated, and stained against NK1.1, CD49b, CD3, NKp46, and IL-22. Representative plots of IL-22 versus NK1.1 expression by NK1.1^+^CD49b^+^NKp46^+^CD3^−^ cells are shown. Results represent three mice/group. **(B)** C57BL/6 mice were infected or not with salivary gland-propagated Smith strain MCMV (5 × 10^4^ pfu). Surface marker expression by pulmonary NK1.1^+^IL-22^+^ and NK1.1^+^IL-22^−^ cells was assessed by flow cytometry. Representative overlay histograms of pulmonary NK1.1^+^IL-22^+^ (dashed purple line) and NK1.1^+^IL-22^−^ (solid blue line) at day 4 postinfection are shown (shaded histogram = FMO control from infected mice). Results are representative of four mice per group.

During acute murine cytomegalovirus (MCMV) infection, T cells, NK T cells, and NK cells produce IL-22 (74). NK cells, which restrict MCMV replication in the spleen, liver, and lung (75, 76), produce IL-22 in response to MCMV infection in the liver and lung but not spleen, demonstrating that IL-22 induction in systemic viral infection is organ-specific (Figure 1A). Similarly, significant IL-22 expression by intrahepatic but not peripheral NK T cells, γδ T cells, and NK cells in hepatitis B virus (HBV)-infected individuals has been demonstrated (77). IL-22-producing NK cells within the peripheral sites of MCMV infection are phenotypically indicative of classical NK cells (Figure 1B). In this infection model, NK cells are stimulated through the activating receptor Ly49H, following recognition of the MCMV m157 protein (78). However, despite expressing significant levels of Ly49H (Figure 1B), mice challenged with m157-deficient (Δm157) MCMV induced comparable pulmonary and hepatic IL-22^+^ NK cell responses to those in WT MCMV infection (Figure 1A). Δm157 and WT MCMV infections also induced comparable early contraction of NK cell responses in the initial phase of infection (76) as indicated by a comparable reduction in NK1.1^+^ cells, as compared to naive mice (Figure 1A). Collectively, experimental data point toward an important function of IL-22 production by NK cells during certain viral infections and suggest a role for cytokines, but not activation receptor ligation in inducing NK cell expression of IL-22.

T cells also produce IL-22 in response to some viruses. Activated T cells isolated from blood of healthy individuals that are repeatedly exposed to human immunodeficiency virus (HIV-1) overproduce IL-22 (79). IL-22-expressing CD4^+^ and CD8^+^ T cells reactive to HIV Gag proteins in uninfected partners of HIV^+^ individuals have been identified (80). T cells isolated from liver and peripheral blood of HBV-infected individuals also express IL-22, as do CD161^+^ CD4^+^ and CD8^+^ T cells enriched in liver of hepatitis C virus (HCV)-infected patients (81, 82). In intestinal rotavirus infection, ILCs are also implicated as a significant source of IL-22 (83, 84), in accordance with the established role of ILCs as IL-22 producers in mucosal tissue [as reviewed in Ref. (85)]. Thus, innate and adaptive antiviral cellular responses can produce IL-22 following viral exposure.

## IL-22 and Protection from Viral Infection and Associated Pathologies

A number of studies have identified or implied an antiviral function for IL-22. IL-22 exerts critical control of rotavirus infection (83, 84) and can cooperate with IL-18 (83) or with IFN-λ (84). In the case of cooperation with IFN-λ, IL-22 augments interferon-stimulated gene (ISG) expression by intestinal epithelial cells (84). In contrast to the dominant induction of STAT3 by IL-22R signaling, IL-22 augmentation of IFN-λ ISG expression in response to rotavirus infection is dependent upon STAT1 activation (84).

Interleukin-22 receptor signaling can induce the expression of chemokines thereby orchestrating recruitment of immune cell subsets to sites of infection. In MCMV infection, IL-22 has a protective role in the lung and liver, where it recruits antiviral neutrophils via induction of CXCL1 (74). In contrast, IL-22 does not influence MCMV replication or neutrophil recruitment in the spleen, suggesting that the influence of IL-22 in a viral infection may depend upon the tissue microenvironment and/or IL-22-responsive cells (74). Chemokine-inducing properties of IL-22 may also be important in the induction of virus-specific antibody responses. Direct cannulation of replication-deficient adenovirus into murine salivary glands induces formation of tertiary lymphoid organs and autoantibody production. This process is dependent upon IL-22 and is associated with IL-22-mediated induction of CXCL13 and CXCL12 (86). Although studies from our own lab using the MCMV infection model demonstrate no protective function of IL-22 in orchestrating T cell-dependent control of virus replication within the salivary glands (unpublished data), it is conceivable that IL-22 may afford mucosal protection from viral infections through the induction of local T:B cell aggregates and virus-specific antibody generation.

Current evidence suggests that IL-22 may exhibit antiviral activity in HIV-infected individuals. IL-22 stimulates production of acute-phase serum amyloid A, which can induce phosphorylation and downregulation of CCR5 expression on immature DCs, thus decreasing susceptibility to HIV-1 infection (79). Also, high systemic levels of IL-22 in Indian individuals infected with HIV-1 subtype C is associated with low viral replication *in vitro*, which was attributed to IL-22 interacting with IL-10 and C-reactive protein (87). Furthermore, loss of IL-22-producing CD4^+^ T cells during chronic HIV infection has been associated with increased damage to the gut epithelium and microbial translocation (88), although IL-22-producing ILCs may compensate for the loss of IL-22^+^ CD4^+^ T cells and maintain mucosal integrity (89). Irrespective of the cellular source of IL-22, systemic IL-22 levels negatively correlate with plasmatic lipopolysaccharide, an indicator of microbial translocation from the gut (87). Thus, IL-22R signaling may also be beneficial in HIV-infected individuals by maintaining barrier function.

In a number of viral infections, IL-22 signaling in the liver provides protection against virus-induced pathology without actually influencing virus replication. IL-22 produced by CD4^+^ T cells is cytoprotective during lymphocytic choriomeningitis virus infection in mice, where it reduces the development of hepatitis (90). In accordance, during HBV infection, IL-22-expressing cells co-localize with liver progenitor cells, and IL-22 promotes STAT3-dependent liver stem/progenitor cells (LPC) proliferation (91). IL-22 also restricts hepatic damage and inflammation induced by dengue virus, a phenotype associated with the suppression of the IL-17R pathway (92). IL-22 is also important for the regeneration of tracheal and lung epithelial cells after influenza infection, preventing lung pathology and secondary bacterial infection (93–95). IL-22^−/−^ mice exhibit impaired regeneration of tracheal epithelium and exacerbated weight loss after clearance of influenza infection; a phenotype rescued by transfer of IL-22-proficient but not deficient NK cells (93). Finally, IL-22 restricts myocardial fibrosis induced by coxsackie virus infection (96), demonstrating the importance for IL-22 restriction of tissue damage and pathology that occurs as a consequence of viral infections.

## IL-22 as a Contributory Factor in Viral Pathogenesis

In certain contexts, IL-22 is harmful to virus-infected hosts. As observed in MCMV infection, IL-22 promotes recruitment of neutrophils in response to West Nile virus (WNV) infection. However, in contrast to the antiviral role for neutrophils in MCMV infection, IL-22-induced neutrophil responses in the central nervous system (CNS) during WNV infection lead to exacerbated pathology and mortality (97). Rather than exhibiting antiviral activity, here neutrophils act as vehicles for WNV dissemination into the CNS, thus aiding the establishment of infection and subsequent inflammation within this site (97).

Intriguingly and in contrast to studies describing a cytoprotective role for IL-22 in tissue repair following HBV infection (91), in a murine model of HBV infection IL-22 supported virus-driven inflammation and consequential liver damage without affecting virus replication. In this model, IL-22 promoted chemokine expression and the recruitment of inflammatory leukocytes (98). Similarly, IL-22 promotes HBV-induced pathology through chemokine-mediated recruitment of T_H_17 cells (77), thus demonstrating that IL-22 can mediate paradoxical tissue-protective and proinflammatory functions in response to the same viral pathogen. HBVs and HCVs are implicated in the development of certain cancers [reviewed in Ref. (99)]. IL-22 protein levels in serum of patients with HBV- and HCV-associated hepatocellular carcinomas is an indicator of poor prognosis (100, 101), implying that virus-induced IL-22 may promote tumor development associated with these infections. Clearly, the possible role that IL-22 plays in the development of other virus-associated cancers (e.g., human papilloma virus-induced cervical cancer) requires a better understanding.

## Is IL-22 a Potentially Useful Therapeutic Target in Viral Infection?

**Figure 2:**
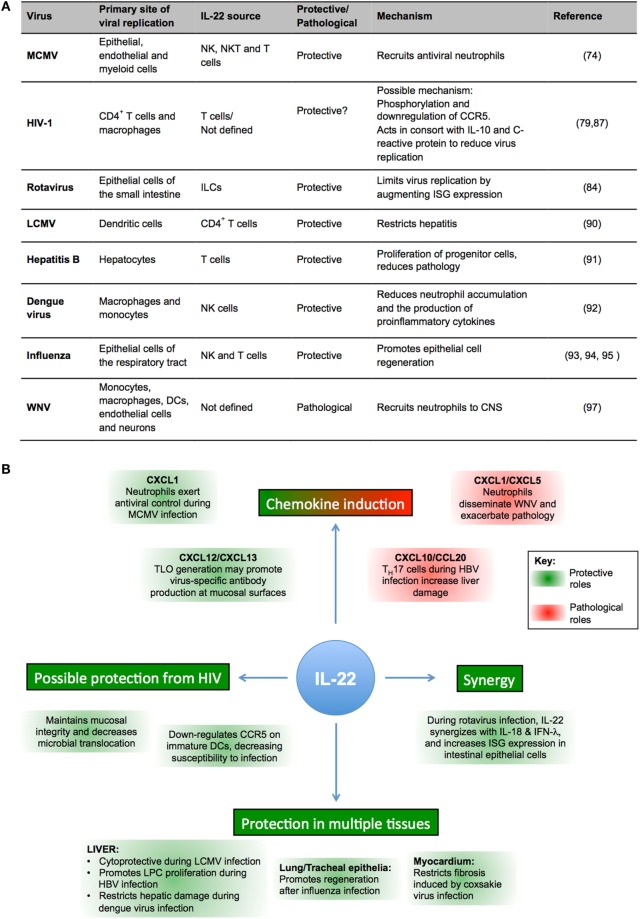
**IL-22 in viral infections**. **(A)** Summary of impact of IL-22 in experimental viral infections. **(B)** Schematic representation of protective (green) and pathological (red) functions of IL-22 in viral infections.

Overall, current data suggest that IL-22 may have an important role in a number of virus infections (as summarized in Figures 2A,B). However, the broad and sometimes paradoxical protective and proinflammatory functions exhibited by IL-22 highlights the complex nature of this cytokine. Thus, does IL-22 represent a useful therapeutic target for clinical intervention strategies for viral infections, and can we predict how IL-22 will influence an immune response induced by a particular virus?

Certain paradigms emerge from published data that may guide future studies that aim to answer these questions. First, it is clear from numerous experimental models that an important function of IL-22 is to induce chemokine expression. The differing roles for neutrophils in MCMV and WNV infections highlight the importance of using *in vivo* infection models to understand what chemokines are induced by IL-22 in response to a particular viral infection and to define the role that IL-22-recruited cells play in antiviral immune responses in infected tissues. Studies of rotavirus infection highlight the importance of IL-22 synergy with other cytokine pathways (83, 84). The timing and context in which IL-22 is produced may greatly influence the impact that this cytokine has on antiviral immune responses.

Understanding the cytokine signature induced by a virus in a particular tissue in addition to defining signaling pathways induced by combinations of cytokines that include IL-22 will likely yield useful information required for predicting the impact of IL-22 in a particular viral infection. Indeed, pathological consequences of IL-22R signaling in HBV infection appear to be intrinsically related to virus-induced IL-17 production (77, 98). Furthermore, IL-22 is implicated in fatal alphavirus encephalomyelitis when unregulated T_H_17 development occurs in the absence of IL-10-mediated suppression (102). Although IL-17 and IL-22 mediate synergistic protective immunity in bacterial infection (55, 103, 104), the combination of these cytokines in viral infections may have pathological consequences.

Should a more defined role for IL-22 during viral infections be elucidated, manipulating the production/availability of IL-22 could prove therapeutically beneficial in treatment and, possibly, prevention of viral-associated disease. For example, administration of IL-22 may accelerate patient recovery from influenza or cytomegalovirus infections by improving lung barrier function or innate antiviral immune responses, respectively. Conversely, IL-22 neutralization could ameliorate virus-induced inflammation in certain infections. However, alteration of IL-22 signaling could have undesired consequences. Blocking the action of IL-22 could increase host susceptibility to bacterial and fungal infection. Conversely, given the protumoral role for T cell-expressed IL-22 in cancer (45, 46, 49, 52, 53, 68), prolonged therapeutic administration IL-22 has the risk of promoting tumor development. Furthermore, more information regarding the mechanisms that regulate IL-22 production by virus-specific T cells is essential before IL-22-inducing vaccines and other therapies are considered for clinical utility.

Given the established role of IL-22 in bacterial infections, the interaction between viruses and bacteria should also be considered when manipulating IL-22 in virus-infected individuals. Antibiotic treatment impairs the induction of protective immunity during influenza infection (105), suggesting that IL-22 modulation of the microbiota may impact on patient outcome. Furthermore, bacterial coinfections cause pathogenesis in individuals infected with viruses such as influenza (106). Thus, diagnosis of bacterial coinfections in patients and understanding how IL-22 impacts on these bacteria will be an important consideration in IL-22-based clinical intervention strategies.

Thus, overall, although IL-22 is clearly an important cytokine in antiviral immune responses, more information regarding the context-dependent nature of IL-22 regulation and function is required before manipulation of this cytokine can be considered in the treatment of virus-infected individuals.

## Ethics Statement

All animal research was performed under the UK Home Office-approved Project License PPL 30/2969, awarded to Ian Humphreys.

## Author Contributions

SGB and MAS performed experiments. SGB, MAS and IRH interpreted the data. AJR generated key reagents and edited the manuscript. SGB, GS, MAS and IRH wrote the manuscript.

## Conflict of Interest Statement

The authors declare that the research was conducted in the absence of any commercial or financial relationships that could be construed as a potential conflict of interest.
